# The next great challenge

**DOI:** 10.1016/S1808-8694(15)30536-X

**Published:** 2015-10-19

**Authors:** Silvio Caldas Neto

A new year is just around the corner, as always bringing new challenges for all of us. Our journal is no exception. However, this time the challenge is even greater. The new CAPES classification for journals, which in principle would not trouble any national journal, has changed its assessment criteria for graduate programs coupled to a new classification system for Brazilian journals, especially those from surgical medical fields, stretching demands farther than we had antecipated. Standards have risen, and risen too much. It is important to raise the bar on standards, push us to reach a higher level of efficiency and excellence. Nonetheless, too high a demand may force us to give up. So much so that it was published in the State of São Paulo newspaper on July 6, 2009, under the title “Ranking Places Brazilian Scientific Journals Under Risk of Extinction”. In a formidable editorial published in the Clinics Journal (2009; 64(8):721-4) its Chief Editor - Mauricio Rocha e Silva even suggests the motto “Down with the New Qualis! Bring Back Realism!” and aptly shows the mismatch between what he calls CAPES exercise of numerology and the reality (or different realities) faced by science in Brazil. It sure is worth checking out.

Back to our talk at the beginning, by itself, without the changes in the criteria for graduate programs, the New Qualis would not be troublesome. At first glance, our journal which went from A (National) to B3 would have its grade lowered. However, it is not that simple. Let me explain: imagine that people would have their heights classified into A, B and C. Class A would group those individuals taller than 2m; B would be those between 1.5 and 2m and C would be those below 1.5m. Now, let's say that at a given time they decide to change this classification to A, B, C and D. Class A would be those individuals taller than 2.5m; B from 2m to 2.5m; C from 1.5 to 1.99m and D those shorter than 1.5m. Now, those individuals who were 1.6m tall and before were class B became class C. This does not mean they became shorter in size, just that they were assigned a different letter, which represents the same height range they had before, with another letter. The problem will only surface if they decide that those individuals from class B or higher would have rights to a certain privilege. Well, that is exactly what is happening to Brazilian scientific journals, especially the medical ones.

Therefore, our journal did not drop in classification. It was Medline material and continues being so. Nonetheless, to be Medline material alone is no longer attractive to the eyes of our graduate programs, which need to maintain a good standard for CAPES. And I repeat: the standards have risen beyond what is reasonable.

Obviously, Brazilian Medical Science can not bend facing such long horizons. It is quite the contrary. We are sure bound for troubled waters; nonetheless, as it happened during other troubled times, it is also certain that we will overcame this new and huge challenge. Our battle now happens in two fronts: one trying to join forces against a journal classification system and programs light years ahead of our reality; and the other is in search of the best quality possible. If ISI is what they want, it is ISI we'll give'em!

